# Associations of plasma clusterin and Alzheimer’s disease-related MRI markers in adults at mid-life: The CARDIA Brain MRI sub-study

**DOI:** 10.1371/journal.pone.0190478

**Published:** 2018-01-11

**Authors:** Thaddeus Haight, R. Nick Bryan, Osorio Meirelles, Russell Tracy, Myriam Fornage, Melissa Richard, Ilya Nasrallah, Kristine Yaffe, David R. Jacobs, Cora Lewis, Pamela Schreiner, Stephen Sidney, Christos Davatzikos, Lenore J. Launer

**Affiliations:** 1 Laboratory of Epidemiology and Population Sciences, National Institute on Aging, Bethesda, MD United States of America; 2 Department of Radiology, University of Pennsylvania Health System, Philadelphia, PA United States of America; 3 Departments of Pathology and Laboratory Medicine, and Biochemistry, University of Vermont College of Medicine, Burlington, VT United States of America; 4 Institute of Molecular Medicine, Research for Human Genetics, University of Texas, Houston, TX United States of America; 5 Department of Psychiatry, University of California-San Francisco, San Francisco, CA United States of America; 6 Division of Epidemiology and Community Health, University of Minnesota, Minneapolis, MN United States of America; 7 Department of Medicine, Division of Preventive Medicine, University of Alabama, Birmingham, AL United States of America; 8 Kaiser Permanente, Division of Research, Oakland, CA United States of America; Johns Hopkins School of Medicine, UNITED STATES

## Abstract

**Background:**

Clinical and epidemiological studies of older persons have implicated clusterin in Alzheimer's disease (AD) pathogenesis. In the context of identifying early biomarkers of risk, we examined associations of plasma clusterin and characteristics of AD in middle-aged individuals from the community.

**Materials and methods:**

Subjects were 639 cognitively normal individuals (mean age 50 ± 3.5) from the Coronary Artery Risk Development in Young Adults (CARDIA) Brain MRI sub-study. Clusterin was quantified using ELISA (mean 255± 31 ng/ml). Associations were assessed between clusterin and volumes of brain regions known to atrophy in early AD, including entorhinal cortex (ECV), hippocampus (HV), and medial temporal lobe (MTLV) volumes (cm^3^). Total brain volume (TBV) and volumes of structures affected in later AD were examined for comparison.

**Results:**

In multivariable models, higher clusterin had a negative non-linear association with ECV (combined left and right hemispheres), and this association was influenced by the highest clusterin levels. Compared to mean clusterin, 1 and 2 standard deviation (SD) level increases in clusterin were associated with -2.1% (95% CI: -3.3,-0.9) and -7.3% (95% CI: -11.3,-3.3) lower ECV, respectively. Similar relationships were observed between clusterin and HV, although the relationship was stronger for left-side HV than the right-side. However, the association was not significant after adjusting for covariates. Negative non-linear associations between clusterin and MTLV were strongest for the left side: compared to mean clusterin, 1 and 2 SD level increases in clusterin were associated with -0.9% (95% CI: -1.9, 0.1) and -3.7% (95% CI: -7.1, -0.3) lower MTLV. There were no significant associations between clusterin and brain structures affected in later AD.

**Conclusions:**

In middle-aged adults unselected for AD, plasma clusterin was associated with lower volume of the entorhinal cortex, an area that atrophies early in AD. Clusterin could be informative as part of a multi-component preclinical marker for AD.

## Introduction

Efforts to develop early and clinically feasible biomarkers of Alzheimer’s disease (AD) are ongoing. Clusterin represents a chaperone protein that is involved in multiple regulatory and physiologic processes. Some of these processes include lipid metabolism, cell apoptosis, and amyloid-β (Aβ) peptide binding[1], which have been implicated in AD and have led to interest in the role of clusterin in AD pathogenesis. Studies of the interaction of clusterin and Aβ, for example, have found that clusterin contributes to Aβ plaque formation as well as modulates Aβ neurotoxicity[2,3]. Moreover, clusterin has been found to be associated with adipose and inflammatory markers, as well as measures of metabolic syndrome, which contribute to AD risk[4–9]. Additionally, separate GWAS studies have identified the gene that encodes clusterin (*CLU*) as a genetic risk factor for AD[10–12]. Therefore, several lines of evidence suggest the potential involvement of clusterin in different pathways to AD.

Clinical studies of patients with mild cognitive impairment (MCI) and AD have found higher levels of plasma clusterin in cases compared to controls, and associations between higher clusterin and smaller tissue volumes in multiple brain areas, particularly the entorhinal cortex and hippocampus, which are known to change with AD. In addition, higher clusterin was related also to severity of cognitive impairment[13,14]. Another study showed increased rates of entorhinal and hippocampal atrophy in individuals with both high CSF clusterin levels and low CSF-Aβ_1–42_ –a pathologic marker of AD[15]. In an epidemiologic study of older adults (mean age 72 years), higher clusterin was associated with prevalent AD, but not with incident AD[16].

In the context of identifying early markers of AD, we examined associations between plasma clusterin and AD-related volume measures in middle-aged adults. The CARDIA Brain MRI sub-study represents a cohort of adults in mid-life with brain MRI, clusterin, genetic, cognitive, and cardiometabolic measures. We hypothesized that early in the disease course that higher clusterin would be associated with lower volumes of structures known to be susceptible in early AD, including the entorhinal cortex, hippocampus, and medial temporal lobe[17–20]. Conversely, we hypothesized that higher clusterin would not be associated with volumes of brain structures that show measurable structural change later in AD.

## Materials and methods

### Study participants

Participants were enrolled in the Coronary Artery Risk Development in Young Adults (CARDIA) Study, a bi-racial longitudinal study to investigate the determinants and development of cardiovascular disease in young adults. Details with respect to recruitment have been previously reported[21]. Of the 5115 adults enrolled in the study, 3499 were evaluated at the 25-year follow-up exam. As part of this exam, a sub-sample was invited to participate in the CARDIA Brain MRI sub-study. The MRI sub-study recruited participants from 3 of the 4 CARDIA field sites. All participants were eligible for inclusion in the sub-study except those with a contraindication to MRI or a body size too large for the MRI scanner. Exclusion criteria were applied at the time of sample selection, or at the MRI site. A total of 719 subjects obtained MRI scans.

All participants provided written informed consent at each CARDIA exam, and institutional review boards (IRB) from each field center approved the study. Separate written consents were obtained for the CARDIA Brain sub-study, genetic study, and serum collection/analysis. The IRB for Intramural Research at the National Institute on Aging (NIA) reviewed and approved the current study.

### MRI acquisition and processing

MRI scans were obtained for patients using 3-T MR scanners located close to each CARDIA clinical site. Details of the scanners used, training of MRI technologists at the different sites, implementation of study protocols, and quality assurance of scanner stability and performance are provided elsewhere[22]. All scans were reviewed for incidental but clinically relevant findings by a radiologist.

Post-scan image processing was performed by the Section of Biomedical Image Analysis (SBIA), Department of Radiology, University of Pennsylvania, as previously described. Briefly, the protocol included control of scan quality and processing through an automated pipeline. Quality checks at initial, intermediate, and final processing steps included visual inspection and identification of outliers based on the distributions of study variables. Of the 719 subjects with MRI scans, 709 had scans that passed inspection.

### MRI measures

An automated computer algorithm segmented MRI structural images of supratentorial brain tissue into gray matter (GM), white matter (WM) and cerebrospinal fluid (CSF) based on previously described methods[23–26]. GM and WM were further characterized as normal and abnormal tissue based on automated analysis of signal intensity, and assigned as 92 anatomic regions of interest (ROIs) covering both hemispheres. These 92 anatomic regions were derived from an expert-delineated atlas[27], which was used as a brain template to which the MRI brain measures were co-registered.

We examined entorhinal cortex (ECV), hippocampal (HV), and medial temporal lobe volumes (MTLV) as early markers of AD given that these smaller brain structures are susceptible to neurofibrillary changes and atrophy in early stages of the disease (i.e., Braak Stages I and II). HV represents the hippocampus proper, excluding the amygdala. MTLV is a composite structure which includes the entorhinal cortex, hippocampus proper, parahippocampal gyrus, and perirhinal cortex. By comparison, total brain volume (TBV), and volumes for total gray matter (TGM) and frontal gray matter (FGM) were examined as larger brain structures for which atrophy occurs at later stages of AD (i.e., Braak Stages V and VI)[17,18]. TBV includes both gray matter and white matter tissue, TGM represents all cerebral gray matter tissue only, and FGM includes frontal lobes, cingulate gyrus, and insula. Given previous reports of asymmetric patterns of atrophy of structures in the different brain hemispheres[28–30], volumes for the left and right sides were examined separately, as well as the combined volumes from both hemispheres.

### Cognitive measures

Cognitive function test scores were recorded for participants for the following: Rey Auditory-Verbal Learning test (RAVLT), Digit Symbol Substitution test (DSST) and Stroop test. RAVLT measures verbal learning and memory and includes different components—i.e., short-term, long-term, and immediate recall[31]. A composite measure based on the average score of these components was generated for further analyses. DSST measures psychomotor performance[32]. Higher scores on the RAVLT and DSST reflect a better cognitive test. STROOP measures executive function and attention[33]. Higher scores on STROOP reflect a worse cognitive test. Scores from all three tests were standardized and z-scores for each were generated. STROOP was categorized as tertiles given it had a skewed distribution.

### Plasma clusterin acquisition and analysis

Sample storage, processing, and protein analysis were performed at the Laboratory of Clinical Biochemistry Research, University of Vermont, Burlington, Vermont. Clusterin was quantified based on solid phase sandwich ELISA (Quantikine Human Clusterin Immunoassay, R&D Systems, Minneapolis, Minnesota). Samples were stored at -80°C in 500 μl tubes and raised to room temperature after 1 freeze-thaw cycle. Standard procedures were applied and 3 control materials were tested in duplicate. Inter-assay CVs were 9.26%, 6.61%, and 6.77%.

Of the participants with MRI data, 646 subjects had serum samples that were assayed, and 641 subjects had quantifiable clusterin values.

### Genetic variants at *CLU* locus

Genotyped and imputed single-nucleotide polymorphism (SNP) data in the *CLU* locus were obtained in a subgroup of the participants (n = 434), including rs11136000, rs2279590, and rs99331888, which have been identified previously in GWAS studies of AD patients[10,11], in addition to other SNPs within the *CLU* locus (chromosome 8 position: 27.452–27.474 Mb). Processing of DNA samples and imputation was performed using procedures described previously[34]. Given DNA sample processing was performed separately for blacks and whites, and given potential population-level differences in genetic and MRI measure associations, we examined data jointly as well as stratified by blacks (n = 113) and whites (n = 321), respectively Details are provided in S1 Appendix.

### Covariates

Covariates examined included age (years) at the 25-year visit, sex, race (black/white), education (<16 years/≥16 years), depression (<16, ≥16 C-ESD), BMI (kg/m^2^), smoking status (0 = never, 1 = former, 2 = current), APOE e4 genotype status (0 = no e4 allele, 1 = ≥ 1 e4 alleles), and high sensitivity C-reactive protein (ug/ml) (hsCRP). Other covariates included a physical activity score[35], prehypertension/ hypertension status, diabetes, dyslipidemia (variables defined as yes/no), based on American Heart Association (AHA) 2012 criteria, and measured at the 25-year visit.

### Statistical analysis

**Fig 1:**
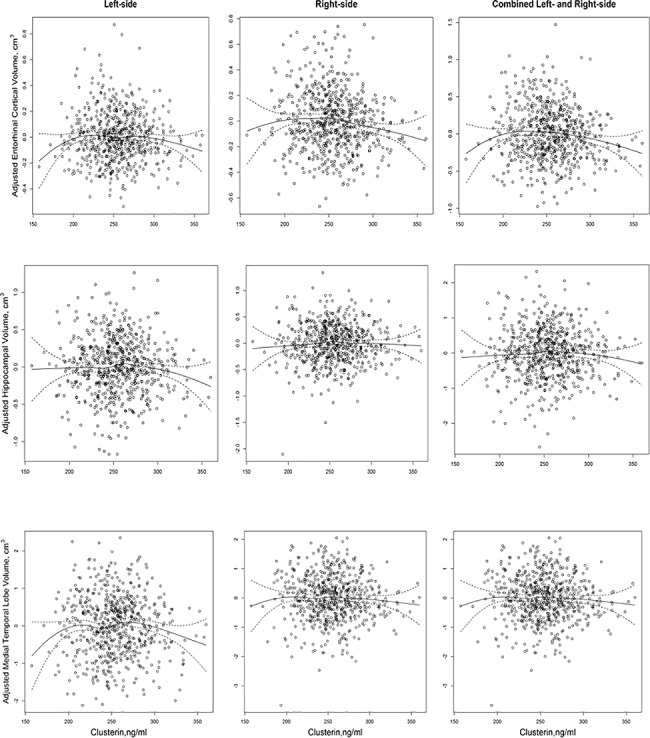
Volume measures of AD-vulnerable brain regions and plasma clusterin. The volume measures include left-side, right-side and combined left and right-side entorhinal cortical volume, hippocampal volume, and medial temporal lobe volume, adjusted for supratentorial brain volume. Plots were fitted with locally weighted smoothing (lowess) functions (95% CI).

We examined associations of clusterin and MRI volume measures for different brain regions. First, we examined clusterin and the different MRI measures graphically, with and without adjustment for supratentorial volume. Locally weighted smoothing (Lowess) functions were applied to examine potential non-linearity in the data (Fig 1).

Of the 641 subjects, 2 subjects with outlying clusterin values (i.e., < 3 SD below group mean) were excluded to avoid potential end-effects.

Given non-linearity between clusterin and the MRI volumes of the smaller structures (i.e., entorhinal cortex, hippocampus, MTL), we applied association models that included linear and quadratic terms for clusterin. F-tests confirmed that the additional quadratic term provided a better model fit (data not shown). Similar models were used to examine the associations of clusterin and the MRI volumes of the larger brain structures.

Clusterin was centered and standardized so that its effects could be interpreted (see S2 Appendix for further details). Interpretation of coefficients for each model is as follows: β_0_ represents the expected MRI volume for mean clusterin (255 ng/ml); β_1_ represents the slope of the association between clusterin and MRI volume at mean clusterin; and β_2_ represents the *change in the slope* of the association between clusterin and MRI volume for each 1 SD difference in clusterin relative to its mean. Fitted estimates were used to determine the relative % difference for the different volume measures for subjects with clusterin levels 1 and 2 SD above mean clusterin, compared to the volume measures in subjects with mean clusterin. Confidence intervals for % differences were based on 1000 bootstrap samples.

We assessed also potential parallel associations between clusterin and different cognitive measures (i.e., DSST, RAVLT, STROOP—see previous ‘Cognitive measures’ section). First, we examined the relationships of clusterin with these different measures graphically (data not shown). For DSST and RAVLT, we examined these as continuous measures with respect to clusterin. Given STROOP was categorized, we examined the log-odds of its distribution (i.e., second vs. first tertile and third vs. first tertile) with respect to deciles of clusterin. Subsequently, we applied association models similar to those used for the brain structures, which included linear and quadratic terms for clusterin.

Models were adjusted age, race, sex, education, supratentorial volume, BMI, hsCRP, and cardiovascular risk factors (i.e., hypertension, diabetes, dyslipidemia, and smoking status). Variables found to alter the association of plasma clusterin and the MRI or cognitive measures were retained in the final models.

In the subset of subjects with genetic data, four SNPs (i.e., rs11136000, rs9331888, rs17466684, rs113644261) associated with the MRI measures (e.g., hippocampal volume) were added to the models of the MRI measures one at a time, with the hypothesis that these would decrease confounding of the clusterin coefficients. Significant results were assessed using two-sided t-tests and an alpha level = 0.05.

Statistical analyses were performed with SAS Version 9.3 and R Version 3.0.1.

## Results

Subjects’ characteristics are presented in Table 1. No significant differences in age, clinical characteristics, and volume of brain structures were observed between study participants and those subjects with MRI but no clusterin except the proportions of men and blacks(data not shown). Plasma clusterin was significantly higher in women, blacks, current smokers, and in those with diabetes, dyslipidemia, and higher hsCRP (Table 2). Although plasma clusterin differed by BMI and hypertension status, there were no statistically significant differences after adjusting these for age, race, and sex. Clusterin levels did not differ by APOE e4 status.

**Table 1:** Characteristics of CARDIA Brain MRI sub-study participants^a^.

N	639
Age mean (SD), y	50 (3.5)
Male, No. (%)	312 (48.8)
Black, No. (%)	250 (39.1)
College Education, No. (%)	313 (49.0)
Smoking, No. (%)	
Former	146 (22.8)
Current	99 (15.5)
APOE e4 status ≥1 allele, No. (%)^b^	180 (30.0)
Depression ≥ 16 C-ESD, No. (%) DSST mean (SD) RAVLT mean (SD) STROOP median [IQR]	87(13.6) 70.2 (15.9) 8.6 (2.6) 20 [16, 26.5]
Physical Activity median [IQR], energy units	308 [149,524]
BMI kg/m^2^, No. (%)	
25–29	225 (35.2)
≥ 30	227 (35.5)
hsCRP median [IQR], ug/ml	1.2 [0.5,2.7]
Prehypertension/Hypertension, No. (%)	235 (36.8)
Diabetes, No. (%)	61 (9.5)
Dyslipidemia, No. (%)	244 (38.2)
Plasma clusterin mean (SD), ng/ml	255(31)
Supratentorial brain volume mean (SD), cm^3^	1212 (134)
Regional Brain Volumes mean (SD), cm^3^	
*Small Structures*	
Entorhinal Cortex Left	1.1 (0.2)
Right	1.5 (0.3)
Combined	2.7 (0.4)
Hippocampus Left	3.7 (0.4)
Right	3.2 (0.4)
Combined	6.9 (0.8)
Medial Temporal Left Lobe Right	8.6 (1.1) 8.5 (1.0)
Combined	17.1 (2.0)
*Large Structures*	
Total Brain	986 (107)
Total Gray Matter	519 (54)
Frontal Gray Matter	198 (22)

Abbreviations: APOE e4, apolipoprotein e4; hsCRP, high sensitivity C-reactive protein; EU, energy units; RAVLT, Rey Auditory-Verbal Learning test; DSST, Digit Symbol Substitution test.

^a^Range of missing values 0–6 for most characteristics, DSST (n = 10), RAVLT (n = 13), and STROOP (n = 11).

^b^APOE e4 status was based on 600 subjects.

**Table 2:** Mean differences in plasma clusterin for different study covariates.

Study Covariates	Mean difference in plasma clusterin, ng/ml (95% CI)	*P* value	Adjusted mean difference in plasma clusterin, ng/ml (95% CI)^a^	*P* value
Age, per year	0.5 (-0.2,1.2)	0.18	NA	
Male, yes vs no	-9.9 (-14.8,-5.2)	<0.001	NA	
Black, yes vs no	6.7 (1.8, 11.7)	0.008	NA	
College Education, yes vs no	-3.9 (-8.8, 0.9)	0.11	-3.3 (-8.5,1.8)	0.21
Smoking				
Former vs None	2.9 (-3.1,8.7)	0.35	2.2 (-3.7, 8.1)	0.47
Current vs None	8.1 (1.2,15.0)	0.021	7.6 (0.7, 14.4)	0.030
APOE e4 Status, ≥1 allele vs none	0.1 (-5.2,5.5)	0.96	-0.7 (-6.0, 4.6)	0.79
Depression, ≥ 16 vs <16 C-ESD	5.7 (-1.3,12.8)	0.11	4.0 (-3.0,11.0)	0.26
Physical Activity, per 100 EU	-1.0 (-1.9,-0.1)	0.036	-0.4 (-1.3,0.4)	0.31
BMI				
25–29 vs < 25 kg/m^2^	-3.7 (-9.8, 2.3)	0.22	-2.6 (-8.6,3.4)	0.39
≥ 30 vs < 25	5.7 (-0.3, 11.7)	0.064	4.9 (-1.1, 10.9)	0.11
hsCRP, per ug/ml	1.1 (0.4,1.7)	0.001	0.8 (0.2, 1.5)	0.012
Hypertension, yes vs no	5.1 (0.1,10.1)	0.047	3.8 (-1.3,9.0)	0.14
Diabetes, yes vs no	10.9 (2.7,19.1)	0.009	9.4 (1.3, 17.6)	0.023
Dyslipidemia, yes vs no	7.3 (2.3, 12.2)	0.004	7.7 (2.8, 12.5)	0.002

Abbreviations: APOE e4, apolipoprotein e4; hsCRP, high sensitivity C-reactive protein; EU, energy units.

^a^Adjusted differences from multivariable regression models which included age, sex, race.

**Fig 2:**
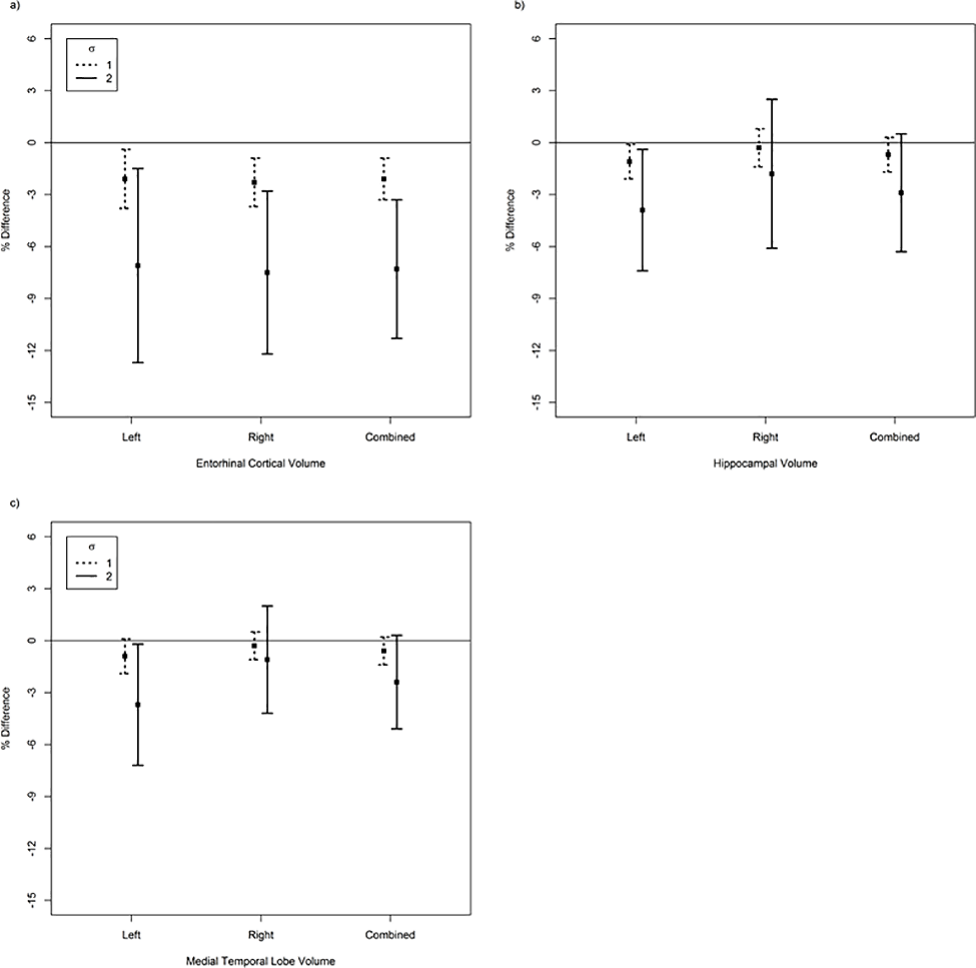
**A-C. Adjusted mean volume differences (95% CI) of smaller brain regions per differences in plasma clusterin levels.** Volumes differences in brain regions estimated for plasma clusterin 1 and 2 SD (σ) above mean plasma clusterin, relative to volumes in subjects with mean plasma clusterin. Differences in volume measures were estimated for left, right, and combined left and right entorhinal cortex (a), hippocampus (b), and medial temporal lobe (c).

Higher plasma clusterin was associated with lower volumes of the smaller brain structures, but results differed for the different structures (Table 3). For example, lower ECV was observed for the left and right hemispheres, and both volumes combined. After adjusting for covariates, only a non-linear association of higher clusterin and lower combined (total) ECV remained significant. Based on the fully-adjusted model, compared to subjects with mean clusterin, subjects whose clusterin was 1 SD higher than the mean had a -2.1% (95% CI: -3.3, -0.9) lower expected ECV, and those whose clusterin was 2 SD higher had a -7.3% (95% CI: -11.3, -3.3) lower expected ECV (Fig 2A).

**Table 3:** Associations of plasma clusterin and MRI volumes for smaller brain structures^a^.

	Left-side			Right-side			Combined		
	Coefficient (95% CI) *P* value			Coefficient (95% CI) *P* value			Coefficient (95% CI) *P* value		
*Unadjusted* *Models*									
ECV Intercept	1.143	(1.121, 1.165)	<0.001	1.549	(1.524, 1.574)	<0.001	2.691	(2.650, 2.732)	<0.001
Clusterin	-0.014	(-0.032, 0.004)	0.12	-0.032	(-0.054, -0.010)	0.003	-0.046	(-0.079, -0.013)	0.009
Clusterin^2^	-0.029	(-0.053, -0.005)	0.012	-0.036	(-0.063, -0.009)	0.010	-0.065	(-0.111, -0.020)	0.004
HV Intercept	3.678	(3.639, 3.717)	<0.001	3.242	(3.203, 3.281)	<0.001	6.920	(6.846, 6.994)	<0.001
Clusterin	-0.024	(-0.057, 0.009)	0.17	-0.005	(-0.038, 0.028)	0.78	-0.028	(-0.091, 0.035)	0.38
Clusterin^2^	-0.053	(-0.096, -0.010)	0.018	-0.039	(-0.082, 0.004)	0.069	-0.092	(-0.172, -0.012)	0.027
MTLV Intercept	8.694	(8.596, 8.792)	<0.001	8.566	(8.470, 8.662)	<0.001	17.261	(17.081, 17.441)	<0.001
Clusterin	-0.064	(-0.146, 0.018)	0.12	-0.081	(-0.161,-0.001)	0.047	-0.146	(-0.297, 0.005)	0.061
Clusterin^2^	-0.150	(-0.256, -0.044)	0.006	-0.089	(-0.193, 0.015)	0.093	-0.240	(-0.438, -0.042)	0.018
*Adjusted* *Models*^b^													
ECV Intercept	1.119	(1.092, 1.146)	<0.001	1.535	(1.502, 1.568)	<0.001	2.654	(2.603, 2.705)	<0.001
Clusterin	-0.003	(-0.019, 0.013)	0.74	-0.014	(-0.032, 0.004)	0.14	-0.016	(-0.043, 0.011)	0.25
Clusterin^2^	-0.019	(-0.039, 0.001)	0.058	-0.022	(-0.046, 0.002)	0.062	-0.041	(-0.076, -0.006)	0.025 ^c^
HV Intercept	3.742	(3.687, 3.797)	<0.001	3.306	(3.251, 3.361)	<0.001	7.048	(6.946, 7.150)	<0.001
Clusterin	-0.007	(-0.036, 0.022)	0.64	0.012	(-0.017, 0.041)	0.42	0.005	(-0.050, 0.060)	0.87
Clusterin^2^	-0.033	(-0.072, 0.006)	0.089	-0.020	(-0.057, 0.017)	0.28	-0.054	(-0.125, 0.017)	0.133
MTLV Intercept	8.802	(8.684, 8.920)	<0.001	8.647	(8.533, 8.761)	<0.001	17.450	(17.244, 17.656)	<0.001
Clusterin	0.003	(-0.060, 0.066)	0.92	-0.006	(-0.067, 0.055)	0.85	-0.003	(-0.113, 0.107)	0.96
Clusterin^2^	-0.083	(-0.163, -0.003)	0.045 ^c^	-0.022	(-0.100, 0.056)	0.56	-0.105	(-0.246, 0.036)	0.15

Abbreviations: ECV, entorhinal cortical volume; HV, hippocampal volume; MTLV, medial temporal lobe volume; hsCRP, high sensitivity C-reactive protein

^a^Plasma clusterin was centered and standardized so that the beta coefficients from the models represent the following: ‘Intercept’represents the mean MRI volume indicated (left column) when clusterin is equal to its mean; ‘Clusterin’ represents the slope of the association between clusterin and the MRI volume at mean clusterin; and ‘Clusterin2’ represents the change in the slope of the association between clusterin and MRI volume for each 1 SD difference in clusterin relative to its mean (see S2 Appendix for further details).

^b^Adjusted for age, sex, race, supratentorial brain volume, hsCRP.

^c^Adjusted results *P* value <0.05

By comparison, higher clusterin was associated with lower HV, where the magnitude of the association was larger for left-side HV than the right, based on the unadjusted model. After adjusting for covariates, however, none of the associations between clusterin and HV remained significant. A similar pattern was observed for MTLV with respect to left and right-side differences. As was the case for HV, results were attenuated after adjusting covariates, although a non-linear association of clusterin and left-side MTLV remained marginally significant. Based on this model, compared with subjects with mean clusterin, subjects whose clusterin was 1 SD higher than the mean had a -0.9% (95% CI: -1.9, 0.1) lower expected MTLV, and those whose clusterin was 2 SD higher had a -3.7% (95% CI: -7.1,-0.3) lower expected MTLV (Fig 2C). Although the association of clusterin and MTLV was larger for the left-side compared to the right, the difference was not statistically significant (p<0.10).

In the analyses of plasma clusterin and larger brain structures, higher plasma clusterin was associated with lower volumes of these structures, although the differences in expected volume at the higher clusterin levels were small (Table 4). However, after adjustment for covariates, these associations were attenuated and none were statistically significant.

**Table 4:** Associations of plasma clusterin and MRI volumes for larger brain regions^a^.

	Unadjusted			Adjusted^b^		
	Coefficient (95% CI) *P* value			Coefficient (95% CI) *P* value		
TBV Intercept	990.7	(980.0, 1000.6)	<0.001	990.5	(986.3, 994.7)	<0.001
Clusterin	-9.1	(-17.4, -0.8)	0.033	-0.9	(-3.2, 1.3)	0.42
Clusterin^2^	-9.8	(-20.6, 1.0)	0.076	-0.6	(-3.5, 2.3)	0.71
TGM Volume Intercept	521.9	(517.0, 526.8)	<0.001	525.7	(522.5, 528.9)	<0.001
Clusterin	-4.8	(-8.9, -0.6)	0.025	-0.6	(-2.3, 1.1)	0.49
Clusterin^2^	-6.7	(-12.1, -1.3)	0.016	-1.8	(-4.0, 0.4)	0.11
FGM Volume Intercept	199.0	(197.0, 201.0)	<0.001	200.1	(198.5, 201.7)	<0.001
Clusterin	-1.8	(-3.4, -0.1)	0.038	-0.3	(-1.2, 0.5)	0.46
Clusterin^2^	-2.3	(-4.5, -0.2)	0.036	-0.5	(-1.6, 0.6)	0.37

Abbreviations: TBV, total brain volume; TGM, total gray matter; FGM, frontal gray matter; hsCRP, high sensitivity C-reactive protein

^a^ Plasma clusterin was centered and standardized so that the beta coefficients from the models represent the following: ‘Intercept’ represents the mean MRI volume indicated (left column) when clusterin is equal to its mean; ‘Clusterin’ represents the slope of the association between clusterin and the MRI volume at mean clusterin; and ‘Clusterin^2^’ represents the change in the slope of the association between clusterin and MRI volume for each 1 SD difference in clusterin relative to its mean (see S2 Appendix for further details).

^b^Adjusted for age, sex, race, supratentorial brain volume, hsCRP.

In subgroup analyses, associations between clusterin and volumes of smaller brain structures did not change after including selected *CLU* SNPs as covariates. In the bi-racial sample (S1–S3 Tables, top rows), the coefficients for clusterin did not differ with inclusion of selected SNPs (Model 1 vs Model 2), but were attenuated after adjusting for the covariates examined in the analysis with all subjects (Model 3). In the stratified analysis, there was an indication in blacks that the association of clusterin and left-side HV, although non-significant, was attenuated with inclusion of selected SNPs (S2 Table–left column, middle rows), while no differences in associations between clusterin and MRI measures were observed for whites (S1–S3 Tables, bottom rows) (Model 1 vs Model 2).

Of the analyses of clusterin and the different cognitive measures, there was a suggestion of an association of higher clusterin and lower DSST that reflected the associations observed between clusterin and the MRI volumes of the smaller brain structures (S4 Table). However, the association was not statistically significant in either the unadjusted analysis or the adjusted analysis. Also, no associations were observed for RAVLT and STROOP with clusterin, respectively.

## Discussion

Our findings indicated associations between plasma clusterin and volumes of brain regions known to atrophy first in AD, with the highest levels of clusterin associated with the lowest volumes in a middle-aged cohort. Associations were strongest between clusterin and ECV, while a marginally significant association was observed for clusterin and left-side MTLV. There was no suggestion of associations between clusterin and volumes of larger brain structures which atrophy at a later stage in AD. While the findings based on these associations were modest, they do suggest a differential impact with respect to higher clusterin and AD-vulnerable brain structures that is consistent with previous evidence[13–15] and early morphologic changes specific to AD[28,36–38].

Our results also revealed associations of higher plasma clusterin with diabetes, dyslipidemia, and higher hsCRP—markers which are known to increase AD risk. Addition of these variables to our models, however, did not substantially alter the results. Similarly, the associations between clusterin and the different MRI measures did not change with the inclusion of different *CLU* SNPs as covariates, although some significant associations between these SNPs and lower HV were observed that were independent of clusterin. Given the limited sample size of the subgroup analysis, it is difficult to conclude whether the observed associations of clusterin and MRI volumes are indeed independent of genetic risk linked to the *CLU* locus. Nonetheless, these findings, and those based on the metabolic and inflammatory markers indicated above, suggest that other processes may contribute to increased clusterin and its observed association with AD-related MRI markers.

Although the observed differences of the brain volumes (i.e., ECV, HV, MTLV) we investigated were small, they are comparable with other studies that have investigated atrophy rates in these regions in AD progression[36–38]. Moreover, we found greater differences in ECV compared to HV, which is a consistent pattern of these studies. In one study of different patient groups > 70 years, for example, greater annual rates of atrophy were observed for ECV compared to HV in AD patients (10.7% vs. 6.0%), patients with cognitive impairment but no AD (4.2% vs. 2.0%), and healthy controls (1.6% vs. 1.0%)[36]. Our results, although based on cross-sectional data and a relatively young cohort, indicated a pattern consistent with morphologic change associated with AD progression.

In addition, we observed different though not statistically significant results between left and right-side HV and MTLV. Other studies have found differences with respect to atrophy patterns between brain hemispheres, with reported changes occurring in the left hemisphere first[28–30]. While the differences we observed were small, they may signal early developments in the disease process. By contrast, the results for ECV were the same regardless of side; therefore, we combined them in order to increase the precision of our estimates.

The fact that we did not observe significant relationships between clusterin and the different cognitive measures that we examined may reflect the relatively young age and the relatively high levels of cognitive functioning of the cohort. It is not surprising that among the different measures, there was a slightly stronger association with DSST, although this was not significant. DSST was found to be the most sensitive measure of cognitive change in a population with high levels of cognition[39]. Previous studies have found that clusterin was related to cognitive impairment, however, these were among subjects with AD[13,16]. Additional follow-up data in the current cohort would allow further assessment of clusterin and its relationship to both structural MRI markers and cognitive markers.

The study has a number of limitations that should be considered. The analysis was cross-sectional, therefore, causal directionality cannot be inferred from the results. Previous reports have found clusterin to be associated with measured atrophy in AD-vulnerable brain regions over time, both in participants with MCI and healthy older adults[13,15]. Follow-up data are needed to assess whether comparable rates of atrophy occur in the current study of adults in mid-life.

Clusterin represents a potentially feasible measure for clinical and research use. However, its measurement in plasma raises questions with regard to its sensitivity, which may be lower in blood relative to the brain, and its specificity as other sources may increase clusterin[4]. Our results indicated a weak but still detectable signal of higher clusterin and lower volumes in AD-vulnerable regions. The fact that the relationship of these measures did not change after adjusting for representative inflammatory and metabolic markers increases confidence with regard to the specificity of the measure. Others have found correlations between CSF and plasma-based clusterin[40], as well as brain tissue based on autopsy data[13], however, more studies are needed that examine the relationship of clusterin in the brain and in plasma.

Another limitation is that we cannot make any inferences with respect to the volume measures investigated and low clusterin levels. It would seem plausible that smaller volumes would be observed for lower clusterin—i.e., some minimal level of clusterin must be present for normal regulatory function and presumably normal brain measures to occur. While the quadratic model would appear to be a good fit at this end of the distribution of clusterin values to evaluate this potential relationship (refer to Fig 1), there are too few data to suggest an association. Additional studies are needed to examine the pattern of association between clusterin and brain volumes, and to assess the extent that lower and higher clusterin may be related to patterns of AD-related brain atrophy.

Despite its limitations, the study has a number of important strengths. To the best of our knowledge, plasma clusterin and AD markers have only been previously assessed in clinical studies or population-based studies of older adults. Given AD-related pathologic changes occur 20 years before the development of clinical symptoms[41], the study sheds light on a critical period early in the AD process. Secondly, the study is a large population-based sample of participants with both plasma clusterin and MRI measures. In addition, the study collected genetic, metabolic, and inflammatory measures which were utilized to ascertain the contributions of other recognized pathophysiologic pathways in AD with respect to the primary study measures. Lastly, the study included MRI volume measures of larger brain structures, which allowed examination of the specificity of our findings with respect to clusterin and smaller MRI structures which are known to be affected first in AD.

In summary, this study examined associations between plasma clusteirn and morphologic markers of early AD in adults in mid-life. Higher clusterin was significantly associated with lower ECV, which represents a brain region that atrophies early in the AD process. Clusterin, a ubiquitous protein involved at many regulatory levels in biological systems, may represent a potentially useful preclinical marker of AD.

## Supporting information

S1 Appendix Genetic variability at *CLU* locus and its association with plasma clusterin and MRI volume measures. (DOC)

S2 Appendix Intepretation of coefficients for quadratic model of plasma clusterin and MRI volumetric measures. (DOC)

S1 Table Associations of *CLU* genetic variants, plasma clusterin, and entorhinal cortical volume. (DOC)

S2 Table Associations of *CLU* genetic variants, plasma clusterin, and hippocampal volume. (DOC)

S3 Table Associations of *CLU* genetic variants, plasma clusterin, and medial temporal lobe volume. (DOC)

S4 Table Associations of plasma clusterin and cognitive measures. (DOC)
