# Supplementary material for: Associations of plasma clusterin and Alzheimer’s disease-related MRI markers in adults at mid-life: The CARDIA Brain MRI sub-study
Source: PLoS One. 2018 Jan 11;13(1):e0190478. doi: 10.1371/journal.pone.0190478 (PMC5764276; doi:10.1371/journal.pone.0190478)
Supplement: S1 Appendix — (DOC) [file pone.0190478.s001.doc]

**S1 Appendix. Genetic variability at *CLU* locus and its association with plasma clusterin and MRI volume measures.**

Study Sample

Genomic data consisting of genotyped and imputed SNPs at the *CLU* locus were available for 321 whites and 113 blacks with MRI and plasma clusterin data. In addition to SNPs which have been identified in previous GWAS studies of patients with AD[10,11], we included SNPs within the *CLU* locus to account for additional variants associated with the *CLU* gene. Specifically, in whites, 56 SNPs were identified. In blacks, 207 SNPs and 13 insertion/deletion regions were identified. In addition, exome data (protein-coding SNPs) were available.

SNP Selection

For purposes of the current analysis, variation in genomic SNP data was examined. None of the SNPs had missing information. We excluded SNPs (137 and 20 for blacks and whites, respectively) with a minor allele frequency (MAF) < 5%, given the limited genetic variation in these SNPs. In addition to SNPs identified previously through GWAS (i.e., rs11136000, rs2279590, rs9331888)[10], we examined other SNPs within the *CLU* locus (chromosome 8 position: 27.452– 27.474 Mb).

Single SNP association analyses with MRI measures

Given potential for correlation between SNPs in the analysis, linkage disequilibrium (LD) was evaluated using available tools (SNAP, Broad Institute). In whites, for example, for each of the 36 SNPs that met MAF, LD was examined with the 35 other SNPs in the list. SNPs were considered in LD if r^2^ > 0.6 or D’ >= 0.8. Upon removing SNPs in LD for each of the SNPs that met MAF, single SNP association analyses were run with: 1) plasma clusterin and 2) different MRI measures as outcome variables, with and without adjustment for covariates. Estimates were obtained of the associations of the SNPs not in LD with the others. Given multiple tests were performed for each of the 36 SNPs, false discovery rate was used to adjust the  p-value for the tests performed. Of the 36 SNPs examined in whites, rs11136000 (e.g., increase in C allele) was associated with lower hippocampal volume (HV), although not significantly (p<0.26), after adjustment for covariates (i.e., age, sex, supratentorial volume, hsCRP) (data not shown). In addition, rs17466684 (e.g., increase in A allele) was weakly associated with lower HV after adjustment for covariates. Other SNPs were similarly associated with lower HV; however, these were in LD with rs11136000 and were excluded from further study. Of the 71 SNPs examined in blacks, rs9331888 (e.g., increase in C allele) was associated with lower left-side HV, although not significantly (p<0.15), after adjustment for covariates, and rs113644261 (i.e., increase in G allele) was significantly associated with higher left-side HV (p<0.04) (data not shown). Different SNPs in blacks were associated with higher clusterin levels, but were not associated with the MRI measures and thus were excluded from further study.

Selection of SNPs as covariates for further investigation

We examined the SNPs above (i.e., rs11136000, rs9331888, rs17466684, rs113644261) in models with plasma clusterin and the MRI volumes of the smaller brain structures (i.e., ECV, HV, MTL), to assess changes in the association between clusterin and the MRI measures. Given SNPs rs11136000 and rs9331888 have been previously identified in GWAS studies, both of these SNPs were examined in blacks and whites together. rs2279590 has been previously identified in GWAS studies of AD also, however this SNP was in LD with rs11136000 in whites, and was not associated with any of the MRI measures in blacks. Both rs17466684 and rs113644261 were associated moderately with HV in whites and blacks, respectively, and were selected for further study in stratified analyses in those groups to determine their effects on the associations between clusterin and MRI volumetric measures.
