# Supplementary material for: Associations of plasma clusterin and Alzheimer’s disease-related MRI markers in adults at mid-life: The CARDIA Brain MRI sub-study
Source: PLoS One. 2018 Jan 11;13(1):e0190478. doi: 10.1371/journal.pone.0190478 (PMC5764276; doi:10.1371/journal.pone.0190478)
Supplement: S2 Appendix — (DOC) [file pone.0190478.s002.doc]

**S2 Appendix.Intepretation of coefficients for quadratic model of plasma clusterin and MRI volumetric measures.**

The quadratic model that relates the different MRI measures and plasma clusterin is as follows: *E* [Y] = β_0_ + β_1_ X + β_2_ X^2^ where X represents plasma clusterin and Y represents MRI volume. To aid in the interpretation of the model coefficients, we performed the following: 1) we centered and standardized clusterin with respect to its mean and standard deviation (i.e., X=0 is mean clusterin); 2) we then assessed the first derivative of the model, where dy represents the expected change in MRI volume and *dx* represents change in clusterin, such that: *dy/dx* = β_1_ + 2β_2 *_ X. Based on this derivation, one interprets β_1_ as the slope (i.e., expected linear change) in MRI volume at the mean clusterin level (X=0), and 2β_2_*X as the expected linear change in MRI volume for each level difference in clusterin (e.g., standardized deviation) relative to its mean; that is, the slope is 2β_2_ at 1 SD above mean clusterin and 4β_2_ at 2 SD above mean clusterin.
