# Supplementary material for: Associations of plasma clusterin and Alzheimer’s disease-related MRI markers in adults at mid-life: The CARDIA Brain MRI sub-study
Source: PLoS One. 2018 Jan 11;13(1):e0190478. doi: 10.1371/journal.pone.0190478 (PMC5764276; doi:10.1371/journal.pone.0190478)
Supplement: S1 Table — (DOC) [file pone.0190478.s003.doc]

| **S1 Table. Associations of *CLU* genetic variants, plasma clusterin, and entorhinal cortical volume ^a,b^** | | | | | | | | | | | | | |
| --- | --- | --- | --- | --- | --- | --- | --- | --- | --- | --- | --- | --- | --- |
|  | Left-side | | | | | Right-side | | | | | Combined | | |
|  | Coefficient (95% CI) *P*-value | | | | | Coefficient (95% CI) *P*-value | | | | | Coefficient (95% CI) *P*-value | | |
| **All participants** |  | | | | |  | | | | |  | | |
| Model 1^c^ Intercept | 1.155 | (1.130, 1.180) | | | <0.001 | 1.562 | (1.533, 1.591) | | | <0.001 | 2.717 | (2.668, 2.766) | <0.001 |
| Clusterin | -0.013 | (-0.035, 0.009) | | | 0.22 | -0.030 | (-0.055, -0.005) | | | 0.020 | -0.043 | (-0.084, -0.002) | 0.038 |
| Clusterin^2^ | -0.025 | (-0.054, 0.004) | | | 0.090 | -0.042 | (-0.075, -0.009) | | | 0.017 | -0.067 | (-0.122, -0.012) | 0.018 |
|  |  |  | | |  |  |  | | |  |  |  |  |
| Model 2^d^ Intercept | 1.139 | (1.090, 1.188) | | | <0.001 | 1.531 | (1.472, 1.590) | | | <0.001 | 2.670 | (2.578, 2.762) | <0.001 |
| Clusterin | -0.013 | (-0.035, 0.009) | | | 0.23 | -0.029 | (-0.054, -0.004) | | | 0.023 | -0.042 | (-0.083, -0.001) | 0.043 |
| Clusterin^2^ | -0.026 | (-0.055, 0.003) | | | 0.087 | -0.042 | (-0.075, -0.009) | | | 0.015 | -0.068 | (-0.123, -0.013) | 0.017 |
| Rs11136000 | 0.013 | (-0.022, 0.048) | | | 0.45 | 0.026 | (-0.015, 0.067) | | | 0.22 | 0.039 | (-0.028, 0.106) | 0.25 |
|  |  |  | | |  |  |  | | |  |  |  |  |
| Model 3^e^  Intercept | 1.103 | (1.052, 1.154) | | | <0.001 | 1.514 | (1.453, 1.575) | | | <0.001 | 2.618 | (2.526, 2.710) | <0.001 |
| Clusterin | -0.003 | (-0.023, 0.017) | | | 0.75 | -0.012 | (-0.034, 0.010) | | | 0.28 | -0.015 | (-0.048, 0.018) | 0.38 |
| Clusterin^2^ | -0.018 | (-0.043, 0.007) | | | 0.15 | -0.032 | (-0.061, -0.003) | | | 0.034 | -0.050 | (-0.095, -0.005) | 0.029 |
| Rs11136000 | 0.014 | (-0.017, 0.045) | | | 0.38 | 0.017 | (-0.018, 0.052) | | | 0.36 | 0.031 | (-0.024, 0.086) | 0.28 |
|  |  |  | | |  |  |  | | |  |  |  |  |
| Model 2 Intercept | 1.178 | (1.113, 1.243) | | | <0.001 | 1.588 | (1.512, 1.664) | | | <0.001 | 2.766 | (2.643, 2.889) | <0.001 |
| Clusterin | -0.014 | (-0.036, 0.008) | | | 0.22 | -0.030 | (-0.055, -0.005) | | | 0.019 | -0.043 | (-0.084, -0.002) | 0.037 |
| Clusterin^2^ | -0.024 | (-0.053, 0.005) | | | 0.10 | -0.041 | (-0.074, -0.008) | | | 0.020 | -0.065 | (-0.120, -0.010) | 0.022 |
| Rs9331888 | -0.016 | (-0.057, 0.025) | | | 0.46 | -0.018 | (-0.067, 0.031) | | | 0.47 | -0.033 | (-0.111, 0.045) | 0.40 |
|  |  |  | | |  |  |  | | |  |  |  |  |
| Model 3 Intercept | 1.135 | (1.076, 1.194) | | | <0.001 | 1.536 | (1.465, 1.607) | | | <0.001 | 2.671 | (2.563, 2.779) | <0.001 |
| Clusterin | -0.003 | (-0.023, 0.017) | | | 0.72 | -0.013 | (-0.035, 0.009) | | | 0.027 | -0.016 | (-0.049, 0.017) | 0.36 |
| Clusterin^2^ | -0.017 | (-0.042, 0.008) | | | 0.17 | -0.031 | (-0.060, -0.002) | | | 0.038 | -0.049 | (-0.094, -0.004) | 0.035 |
| Rs9331888 | -0.011 | (-0.046, 0.024) | | | 0.57 | 0.000 | (-0.043, 0.043) | | | >0.99 | -0.011 | (-0.076, 0.054) | 0.75 |
|  |  |  | | |  |  |  | | |  |  |  |  |
| **Black participants** |  | |  |  | |  | |  |  | |  |  |  |
| Model 1 Intercept | 1.113 | (1.060, 1.166) | | | <0.001 | 1.465 | (1.402, 1.528) | | | <0.001 | 2.578 | (2.478, 2.678) | <0.001 |
| Clusterin | -0.027 | (-0.066, 0.012) | | | 0.19 | -0.050 | (-0.097, -0.003) | | | 0.042 | -0.077 | (-0.153, -0.001) | 0.053 |
| Clusterin^2^ | -0.029 | (-0.098, 0.040) | | | 0.41 | -0.027 | (-0.109, 0.055) | | | 0.51 | -0.056 | (-0.187, 0.075) | 0.41 |
|  |  |  | | |  |  |  | | |  |  |  |  |
| Model 2 Intercept | 1.156 | (1.001, 1.311) | | | <0.001 | 1.574 | (1.390, 1.758) | | | <0.001 | 2.730 | (2.434, 3.026) | <0.001 |
| Clusterin | -0.028 | (-0.069, 0.013) | | | 0.18 | -0.053 | (-0.100, -0.006) | | | 0.032 | -0.081 | (-0.157, -0.005) | 0.041 |
| Clusterin^2^ | -0.027 | (-0.096, 0.042) | | | 0.45 | -0.023 | (-0.105, 0.059) | | | 0.58 | -0.050 | (-0.181, 0.081) | 0.46 |
| Rs9331888 | -0.027 | (-0.117, 0.063) | | | 0.56 | -0.067 | (-0.173, 0.039) | | | 0.22 | -0.094 | (-0.265, 0.077) | 0.29 |
|  |  |  | | |  |  |  | | |  |  |  |  |
| Model 3 Intercept | 1.129 | (0.972, 1.286) | | | <0.001 | 1.563 | (1.391, 1.735) | | | <0.001 | 2.692 | (2.418, 2.966) | <0.001 |
| Clusterin | -0.018 | (-0.057, 0.021) | | | 0.37 | -0.035 | (-0.078, 0.008) | | | 0.10 | -0.053 | (-0.120, 0.014) | 0.13 |
| Clusterin^2^ | -0.046 | (-0.111, 0.019) | | | 0.16 | -0.057 | (-0.128, 0.014) | | | 0.12 | -0.103 | (-0.217, 0.011) | 0.077 |
| Rs9331888 | -0.016 | (-0.100, 0.068) | | | 0.72 | -0.051 | (-0.143, 0.041) | | | 0.28 | -0.066 | (-0.213, 0.081) | 0.38 |
|  |  |  | | |  |  |  | | |  |  |  |  |
| Model 2 Intercept | 1.016 | (0.769, 1.263) | | | <0.001 | 1.315 | (1.023, 1.607) | | | <0.001 | 2.331 | (1.861, 2.801) | <0.001 |
| Clusterin | -0.029 | (-0.070, 0.012) | | | 0.16 | -0.054 | (-0.103, -0.005) | | | 0.030 | -0.083 | (-0.161, -0.005) | 0.037 |
| Clusterin^2^ | -0.024 | (-0.095, 0.047) | | | 0.50 | -0.020 | (-0.102, 0.062) | | | 0.63 | -0.045 | (-0.178, 0.088) | 0.51 |
| Rs113644261 | 0.050 | (-0.073, 0.173) | | | 0.43 | 0.078 | (-0.069, 0.225) | | | 0.31 | 0.127 | (-0.110, 0.364) | 0.30 |
|  |  |  | | |  |  |  | | |  |  |  |  |
| Model 3 Intercept | 0.976 | (0.741, 1.211) | | | <0.001 | 1.274 | (1.015, 1.533) | | | <0.001 | 2.250 | (1.836, 2.664) | <0.001 |
| Clusterin | -0.019 | (-0.058, 0.020) | | | 0.32 | -0.037 | (-0.078, 0.004) | | | 0.086 | -0.056 | (-0.123, 0.011) | 0.10 |
| Clusterin^2^ | -0.042 | (-0.107, 0.023) | | | 0.21 | -0.052 | (-0.123, 0.019) | | | 0.15 | -0.094 | (-0.208, 0.020) | 0.11 |
| Rs113644261 | 0.064 | (-0.052, 0.180) | | | 0.27 | 0.104 | (-0.021, 0.229) | | | 0.11 | 0.168 | (-0.032, 0.368) | 0.10 |
|  |  |  | | |  |  |  | | |  |  |  |  |
| **White participants** |  | | | | | | | | | | | | |
| Model 1 Intercept | 1.168 | (1.011, 1.325) | | | <0.001 | 1.591 | (1.558, 1.624) | | | <0.001 | 2.758 | (2.703, 2.813) | <0.001 |
| Clusterin | -0.005 | (-0.030, 0.020) | | | 0.73 | -0.014 | (-0.043, 0.015) | | | 0.33 | -0.019 | (-0.066, 0.028) | 0.43 |
| Clusterin^2^ | -0.020 | (-0.051, 0.011) | | | 0.22 | -0.037 | (-0.074, 0.000) | | | 0.046 | -0.057 | (-0.116, 0.002) | 0.062 |
|  |  |  | | |  |  |  | | |  |  |  |  |
| Model 2 Intercept | 1.159 | (1.092, 1.226) | | | <0.001 | 1.569 | (1.493, 1.645) | | | <0.001 | 2.729 | (2.604, 2.854) | <0.001 |
| Clusterin | -0.004 | (-0.029, 0.021) | | | 0.74 | -0.014 | (-0.043, 0.015) | | | 0.36 | -0.018 | (-0.065, 0.029) | 0.46 |
| Clusterin^2^ | -0.020 | (-0.051, 0.011) | | | 0.22 | -0.037 | (-0.074, 0.000) | | | 0.045 | -0.057 | (-0.116, 0.002) | 0.061 |
| Rs11136000 | 0.006 | (-0.041, 0.053) | | | 0.79 | 0.017 | (-0.036, 0.070) | | | 0.53 | 0.024 | (-0.064, 0.112) | 0.60 |
|  |  |  | | |  |  |  | | |  |  |  |  |
| Model 3 Intercept | 1.109 | (1.046, 1.172) | | | <0.001 | 1.506 | (1.430, 1.582) | | | <0.001 | 2.615 | (2.499, 2.731) | <0.001 |
| Clusterin | 0.002 | (-0.020, 0.024) | | | 0.87 | -0.005 | (-0.030, 0.020) | | | 0.74 | -0.003 | (-0.042, 0.036) | 0.90 |
| Clusterin^2^ | -0.007 | (-0.034, 0.020) | | | 0.62 | -0.023 | (-0.054, 0.008) | | | 0.17 | -0.029 | (-0.078, 0.020) | 0.24 |
| Rs11136000 | 0.017 | (-0.022, 0.056) | | | 0.39 | 0.028 | (-0.019, 0.075) | | | 0.25 | 0.045 | (-0.026, 0.116) | 0.22 |
|  |  |  | | |  |  |  | | |  |  |  |  |
| Model 2 Intercept | 1.174 | (1.139, 1.209) | | | <0.001 | 1.601 | (1.560, 1.642) | | | <0.001 | 2.775 | (2.706, 2.844) | <0.001 |
| Clusterin | -0.004 | (-0.029, 0.021) | | | 0.73 | -0.014 | (-0.043, 0.015) | | | 0.34 | -0.019 | (-0.066, 0.028) | 0.44 |
| Clusterin^2^ | -0.021 | (-0.052, 0.010) | | | 0.20 | -0.038 | (-0.075, -0.001) | | | 0.038 | -0.060 | (-0.121, 0.001) | 0.052 |
| Rs17466684 | -0.018 | (-0.079, 0.043) | | | 0.56 | -0.030 | (-0.099, 0.039) | | | 0.39 | -0.048 | (-0.162, 0.066) | 0.41 |
|  |  |  | | |  |  |  | | |  |  |  |  |
| Model 3 Intercept | 1.140 | (1.097, 1.183) | | | <0.001 | 1.556 | (1.505, 1.607) | | | <0.001 | 2.696 | (2.618, 2.774) | <0.001 |
| Clusterin | 0.002 | (-0.020, 0.024) | | | 0.88 | -0.005 | (-0.030, 0.020) | | | 0.71 | -0.003 | (-0.042, 0.036) | 0.87 |
| Clusterin^2^ | -0.008 | (-0.035, 0.019) | | | 0.57 | -0.024 | (-0.057, 0.009) | | | 0.15 | -0.032 | (-0.081, 0.017) | 0.20 |
| Rs17466684 | -0.026 | (-0.075, 0.023) | | | 0.31 | -0.038 | (-0.099, 0.023) | | | 0.22 | -0.064 | (-0.156, 0.028) | 0.17 |
| Abbreviations: ECV, entorhinal cortex volume; HV, hippocampal volume; MTLV, medial temporal lobe volume; hsCRP, high sensitivity C-reactive protein.  ^a^ Based on 434 subjects with SNP, plasma clusterin, and MRI data.  ^b^Plasma clusterin was centered and standardized so that the beta coefficients from the models represent the following: ‘Intercept’ represents the mean MRI volume indicated (left column) when clusterin is equal to its mean; ‘Clusterin’ represents the slope of the association between clusterin and the MRI volume at mean clusterin; and ‘Clusterin^2^’ represents the change in the slope of the association between clusterin and MRI volume for each 1 SD difference in clusterin relative to its mean (see S2 Appendix for further details).  ^c^ Model 1: Intercept, clusterin, clusterin^2^.  ^d^ Model 2: Intercept, clusterin, clusterin^2^ and genetic variant (i.e. *Rs11136000*) previously found to be moderately associated with MRI markers in single SNP analysis (see S1 Appendix for further details).  ^e^ Model 3: Model 2 plus additional covariates including age, sex, race (included in non-stratified analysis), supratentorial brain volume, hsCRP. | | | | | | | | | | | | | |
