# Supplementary material for: Associations of plasma clusterin and Alzheimer’s disease-related MRI markers in adults at mid-life: The CARDIA Brain MRI sub-study
Source: PLoS One. 2018 Jan 11;13(1):e0190478. doi: 10.1371/journal.pone.0190478 (PMC5764276; doi:10.1371/journal.pone.0190478)
Supplement: S2 Table — (DOC) [file pone.0190478.s004.doc]

| **S2 Table. Associations of *CLU* genetic variants, plasma clusterin, and hippocampal volume ^a,b^** | | | | | | | | | | | | | |
| --- | --- | --- | --- | --- | --- | --- | --- | --- | --- | --- | --- | --- | --- |
|  | Left-side | | | | | Right-side | | | | | Combined | | |
|  | Coefficient (95% CI) *P*-value | | | | | Coefficient (95% CI) *P*-value | | | | | Coefficient (95% CI) *P*-value | | |
| **All participants** |  | | | | |  | | | | |  | | |
| Model 1^c^ Intercept | 3.707 | (3.660, 3.754) | | | <0.001 | 3.257 | (3.212, 3.302) | | | <0.001 | 6.964 | (6.878, 7.050) | <0.001 |
| Clusterin | -0.022 | (-0.061, 0.017) | | | 0.27 | -0.003 | (-0.042, 0.036) | | | 0.89 | -0.024 | (-0.097, 0.049) | 0.51 |
| Clusterin^2^ | -0.072 | (-0.125, -0.019) | | | 0.008 | -0.036 | (-0.089, 0.017) | | | 0.17 | -0.108 | (-0.206, -0.010) | 0.031 |
|  |  |  | | |  |  |  | | |  |  |  |  |
| Model 2^d^ Intercept | 3.720 | (3.632, 3.808) | | | <0.001 | 3.272 | (3.184, 3.360) | | | <0.001 | 6.992 | (6.827, 7.157) | <0.001 |
| Clusterin | -0.022 | (-0.061, 0.017) | | | 0.27 | -0.003 | (-0.042, 0.036) | | | 0.88 | -0.025 | (-0.098, 0.048) | 0.50 |
| Clusterin^2^ | -0.072 | (-0.125, -0.019) | | | 0.008 | -0.036 | (-0.089, 0.017) | | | 0.18 | -0.108 | (-0.206, -0.010) | 0.032 |
| Rs11136000 | -0.010 | (-0.073, 0.053) | | | 0.75 | -0.013 | (-0.076, 0.050) | | | 0.69 | -0.023 | (-0.141, 0.095) | 0.70 |
|  |  |  | | |  |  |  | | |  |  |  |  |
| Model 3^e^ Intercept | 3.760 | (3.664, 3.856) | | | <0.001 | 3.322 | (3.226, 3.418) | | | <0.001 | 7.081 | (6.905, 7.257) | <0.001 |
| Clusterin | -0.004 | (-0.039, 0.031) | | | 0.81 | 0.012 | (-0.023, 0.047) | | | 0.51 | 0.008 | (-0.057, 0.073) | 0.82 |
| Clusterin^2^ | -0.058 | (-0.105, -0.011) | | | 0.017 | -0.023 | (-0.070, 0.024) | | | 0.33 | -0.081 | (-0.167, 0.005) | 0.065 |
| Rs11136000 | -0.015 | (-0.074, 0.044) | | | 0.60 | -0.017 | (-0.076, 0.042) | | | 0.57 | -0.032 | (-0.138, 0.074) | 0.56 |
|  |  |  | | |  |  |  | | |  |  |  |  |
| Model 2 Intercept | 3.775 | (3.657, 3.893) | | | <0.001 | 3.326 | (3.210, 3.442) | | | <0.001 | 7.101 | (6.881, 7.321) | <0.001 |
| Clusterin | -0.022 | (-0.061, 0.017) | | | 0.26 | -0.003 | (-0.042, 0.036) | | | 0.87 | -0.025 | (-0.098, 0.048) | 0.49 |
| Clusterin^2^ | -0.069 | (-0.122, -0.016) | | | 0.011 | -0.034 | (-0.087, 0.019) | | | 0.21 | -0.103 | (-0.201, -0.005) | 0.041 |
| Rs9331888 | -0.046 | (-0.120, 0.028) | | | 0.22 | -0.048 | (-0.121, 0.025) | | | 0.20 | -0.094 | (-0.231, 0.043) | 0.18 |
|  |  |  | | |  |  |  | | |  |  |  |  |
| Model 3 Intercept | 3.782 | (3.668, 3.896) | | | <0.001 | 3.348 | (3.234, 3.462) | | | <0.001 | 7.130 | (6.924, 7.336) | <0.001 |
| Clusterin | -0.005 | (-0.040, 0.030) | | | 0.78 | 0.011 | (-0.024, 0.046) | | | 0.54 | 0.006 | (-0.059, 0.071) | 0.86 |
| Clusterin^2^ | -0.057 | (-0.104, -0.010) | | | 0.019 | -0.022 | (-0.069, 0.025) | | | 0.35 | -0.079 | (-0.165, 0.007) | 0.072 |
| Rs9331888 | -0.030 | (-0.099, 0.039) | | | 0.39 | -0.034 | (-0.103, 0.035) | | | 0.32 | -0.064 | (-0.187, 0.059) | 0.31 |
|  |  |  | | |  |  |  | | |  |  |  |  |
| **Black participants** |  | |  |  | |  | |  |  | |  |  |  |
| Model 1 Intercept | 3.616 | (3.512, 3.720) | | | <0.001 | 3.168 | (3.068, 3.268) | | | <0.001 | 6.784 | (6.590, 6.978) | <0.001 |
| Clusterin | -0.017 | (-0.095, 0.061) | | | 0.67 | 0.015 | (-0.061, 0.091) | | | 0.70 | -0.003 | (-0.152, 0.146) | 0.97 |
| Clusterin^2^ | -0.049 | (-0.184, 0.086) | | | 0.48 | 0.018 | (-0.115, 0.151) | | | 0.80 | -0.032 | (-0.289, 0.225) | 0.81 |
|  |  |  | | |  |  |  | | |  |  |  |  |
| Model 2 Intercept | 3.920 | (3.618, 4.222) | | | <0.001 | 3.372 | (3.076, 3.668) | | | <0.001 | 7.292 | (6.722, 7.862) | <0.001 |
| Clusterin | -0.026 | (-0.104, 0.052) | | | 0.51 | 0.009 | (-0.067, 0.085) | | | 0.82 | -0.017 | (-0.166, 0.132) | 0.82 |
| Clusterin^2^ | -0.037 | (-0.172, 0.098) | | | 0.59 | 0.026 | (-0.107, 0.159) | | | 0.71 | -0.012 | (-0.267, 0.243) | 0.93 |
| Rs9331888 | -0.188 | (-0.362, -0.014) | | | 0.038 | -0.125 | (-0.297, 0.047) | | | 0.16 | -0.313 | (-0.664, 0.018) | 0.067 |
|  |  |  | | |  |  |  | | |  |  |  |  |
| Model 3 Intercept | 4.074 | (3.790, 4.358) | | | <0.001 | 3.551 | (3.261, 3.841) | | | <0.001 | 7.625 | (7.082, 8.168) | <0.001 |
| Clusterin | -0.011 | (-0.082, 0.060) | | | 0.75 | 0.013 | (-0.058, 0.084) | | | 0.73 | 0.001 | (-0.132, 0.134) | 0.98 |
| Clusterin^2^ | -0.074 | (-0.192, 0.044) | | | 0.22 | -0.012 | (-0.132, 0.108) | | | 0.84 | -0.087 | (-0.310, 0.136) | 0.45 |
| Rs9331888 | -0.221 | (-0.374, -0.068) | | | 0.006 | -0.150 | (-0.307, 0.007) | | | 0.062 | -0.372 | (-0.664, -0.080) | 0.014 |
|  |  |  | | |  |  |  | | |  |  |  |  |
| Model 2 Intercept | 3.001 | (2.527, 3.475) | | | <0.001 | 2.689 | (2.223, 3.155) | | | <0.001 | 5.691 | (4.793, 6.589) | <0.001 |
| Clusterin | -0.034 | (-0.112, 0.044) | | | 0.39 | 0.002 | (-0.074, 0.078) | | | 0.97 | -0.033 | (-0.180, 0.114) | 0.66 |
| Clusterin^2^ | -0.021 | (-0.156, 0.114) | | | 0.76 | 0.039 | (-0.092, 0.170) | | | 0.56 | 0.018 | (-0.237, 0.273) | 0.89 |
| Rs113644261 | 0.317 | (0.078, 0.556) | | | 0.011 | 0.247 | (0.012, 0.482) | | | 0.042 | 0.564 | (0.111, 1.017) | 0.016 |
|  |  |  | | |  |  |  | | |  |  |  |  |
| Model 3 Intercept | 3.006 | (2.581, 3.431) | | | <0.001 | 2.778 | (2.343, 3.213) | | | <0.001 | 5.784 | (4.973, 6.589) | <0.001 |
| Clusterin | -0.014 | (-0.083, 0.055) | | | 0.69 | 0.010 | (-0.061, 0.081) | | | 0.78 | -0.004 | (-0.180, 0.114) | 0.95 |
| Clusterin^2^ | -0.060 | (-0.176, 0.056) | | | 0.32 | -0.001 | (-0.121, 0.119) | | | 0.99 | -0.061 | (-0.282, 0.160) | 0.59 |
| Rs113644261 | 0.355 | (0.149, 0.561) | | | 0.001 | 0.265 | (0.053, 0.477) | | | 0.016 | 0.620 | (0.226, 1.014) | 0.003 |
|  |  |  | | |  |  |  | | |  |  |  |  |
| **White participants** |  | | | | | | | | | | | | |
| Model 1 Intercept | 3.737 | (3.686, 3.788) | | | <0.001 | 3.284 | (3.233, 3.335) | | | <0.001 | 7.021 | (6.925, 7.117) | <0.001 |
| Clusterin | -0.016 | (-0.061, 0.029) | | | 0.47 | -0.003 | (-0.048, 0.042) | | | 0.88 | -0.020 | (-0.102, 0.062) | 0.64 |
| Clusterin^2^ | -0.074 | (-0.129, -0.019) | | | 0.010 | -0.048 | (-0.103, 0.007) | | | 0.087 | -0.122 | (-0.224, -0.020) | 0.020 |
|  |  |  | | |  |  |  | | |  |  |  |  |
| Model 2 Intercept | 3.806 | (3.690, 3.922) | | | <0.001 | 3.391 | (3.275, 3.507) | | | <0.001 | 7.197 | (6.983, 7.411) | <0.001 |
| Clusterin | -0.019 | (-0.064, 0.026) | | | 0.401 | -0.007 | (-0.052, 0.038) | | | 0.75 | -0.026 | (-0.108, 0.056) | 0.53 |
| Clusterin^2^ | -0.073 | (-0.128, -0.018) | | | 0.010 | -0.048 | (-0.103, 0.007) | | | 0.092 | -0.121 | (-0.223, -0.019) | 0.021 |
| Rs11136000 | -0.054 | (-0.136, 0.028) | | | 0.19 | -0.083 | (-0.163, -0.003) | | | 0.044 | -0.138 | (-0.289, 0.013) | 0.073 |
|  |  |  | | |  |  |  | | |  |  |  |  |
| Model 3 Intercept | 3.800 | (3.680, 3.920) | | | <0.001 | 3.404 | (3.284, 3.524) | | | <0.001 | 7.203 | (6.987, 7.419) | <0.001 |
| Clusterin | -0.011 | (-0.052, 0.030) | | | 0.60 | 0.003 | (-0.038, 0.044) | | | 0.88 | -0.008 | (-0.082, 0.066) | 0.84 |
| Clusterin^2^ | -0.052 | (-0.103,-0.001) | | | 0.046 | -0.025 | (-0.076, 0.026) | | | 0.34 | -0.077 | (-0.169, 0.015) | 0.10 |
| Rs11136000 | -0.043 | (-0.117, 0.031) | | | 0.26 | -0.080 | (-0.154, -0.006) | | | 0.034 | -0.123 | (-0.258, 0.012) | 0.073 |
|  |  |  | | |  |  |  | | |  |  |  |  |
| Model 2 Intercept | 3.770 | (3.707, 3.833) | | | <0.001 | 3.317 | (3.254, 3.380) | | | <0.001 | 7.087 | (6.971, 7.203) | <0.001 |
| Clusterin | -0.016 | (-0.061, 0.029) | | | 0.49 | -0.003 | (-0.046, 0.040) | | | 0.91 | -0.018 | (-0.100, 0.064) | 0.66 |
| Clusterin^2^ | -0.079 | (-0.134, -0.024) | | | 0.006 | -0.054 | (-0.109, 0.001) | | | 0.058 | -0.133 | (-0.235, -0.031) | 0.011 |
| Rs17466684 | -0.098 | (-0.204, 0.008) | | | 0.070 | -0.093 | (-0.199, 0.013) | | | 0.082 | -0.191 | (-0.385, 0.003) | 0.054 |
|  |  |  | | |  |  |  | | |  |  |  |  |
| Model 3 Intercept | 3.786 | (3.706, 3.866) | | | <0.001 | 3.341 | (3.261, 3.421) | | | <0.001 | 7.127 | (6.980, 7.274) | <0.001 |
| Clusterin | -0.008 | (-0.049, 0.033) | | | 0.72 | 0.008 | (-0.033, 0.049) | | | 0.71 | 0.000 | (-0.074, 0.074) | >0.99 |
| Clusterin^2^ | -0.059 | (-0.110, -0.008) | | | 0.024 | -0.032 | (-0.083, 0.019) | | | 0.22 | -0.090 | (-0.182, 0.002) | 0.055 |
| Rs17466684 | -0.111 | (-0.207, -0.015) | | | 0.022 | -0.109 | (-0.205, -0.013) | | | 0.025 | -0.221 | (-0.393, -0.049) | 0.012 |
| Abbreviations: ECV, entorhinal cortex volume; HV, hippocampal volume; MTLV, medial temporal lobe volume; hsCRP, high sensitivity C-reactive protein.  ^a^ Based on 434 subjects with SNP, plasma clusterin, and MRI data.  ^b^Plasma clusterin was centered and standardized so that the beta coefficients from the models represent the following: ‘Intercept’ represents the mean MRI volume indicated (left column) when clusterin is equal to its mean; ‘Clusterin’ represents the slope of the association between clusterin and the MRI volume at mean clusterin; and ‘Clusterin^2^’ represents the change in the slope of the association between clusterin and MRI volume for each 1 SD difference in clusterin relative to its mean (see S2 Appendix for further details).  ^c^ Model 1: Intercept, clusterin, clusterin^2^.  ^d^ Model 2: Intercept, clusterin, clusterin^2^ and genetic variant (i.e. *Rs11136000*) previously found to be moderately associated with MRI markers in single SNP analysis (see S1 Appendix for further details).  ^e^ Model 3: Model 2 plus additional covariates including age, sex, race (included in non-stratified analysis), supratentorial brain volume, hsCRP. | | | | | | | | | | | | | |
