# Supplementary material for: Associations of plasma clusterin and Alzheimer’s disease-related MRI markers in adults at mid-life: The CARDIA Brain MRI sub-study
Source: PLoS One. 2018 Jan 11;13(1):e0190478. doi: 10.1371/journal.pone.0190478 (PMC5764276; doi:10.1371/journal.pone.0190478)
Supplement: S3 Table — (DOC) [file pone.0190478.s005.doc]

| **S3 Table. Associations of *CLU* genetic variants, plasma clusterin, and medial temporal lobe volume ^a,b^** | | | | | | | | | | | | | |
| --- | --- | --- | --- | --- | --- | --- | --- | --- | --- | --- | --- | --- | --- |
|  | Left-side | | | | | Right-side | | | | | Combined | | |
|  | Beta (95% CI) *P*-value | | | | | Beta (95% CI) *P*-value | | | | | Beta (95% CI) *P*-value | | |
| **All participants** |  | | | | |  | | | | |  | | |
| Model 1^c^ Intercept | 8.787 | (8.671, 8.903) | | | <0.001 | 8.623 | (8.511, 8.735) | | | <0.001 | 17.410 | (17.200, 17.620) | <0.001 |
| Clusterin | -0.060 | (-0.156, 0.036) | | | 0.22 | -0.061 | (-0.153, 0.031) | | | 0.20 | -0.121 | (-0.295, 0.053) | 0.18 |
| Clusterin^2^ | -0.154 | (-0.285, -0.023) | | | 0.021 | -0.070 | (-0.197, 0.057) | | | 0.28 | -0.225 | (-0.464, 0.014) | 0.066 |
|  |  |  | | |  |  |  | | |  |  |  |  |
| Model 2^d^ Intercept | 8.717 | (8.497, 8.937) | | | <0.001 | 8.554 | (8.342, 8.766) | | | <0.001 | 17.271 | (16.871, 17.671) | <0.001 |
| Clusterin | -0.058 | (-0.154, 0.038) | | | 0.23 | -0.059 | (-0.153, 0.035) | | | 0.21 | -0.118 | (-0.294, 0.058) | 0.19 |
| Clusterin^2^ | -0.155 | (-0.286, -0.024) | | | 0.020 | -0.071 | (-0.196, 0.054) | | | 0.27 | -0.227 | (-0.466, 0.012) | 0.063 |
| Rs11136000 | 0.059 | (-0.098, 0.216) | | | 0.46 | 0.058 | (-0.095, 0.211) | | | 0.45 | 0.117 | (-0.169, 0.403) | 0.42 |
|  |  |  | | |  |  |  | | |  |  |  |  |
| Model 3^e^ Intercept | 8.788 | (8.580, 8.996) | | | <0.001 | 8.598 | (8.396, 8.800) | | | <0.001 | 17.387 | (17.030, 17.744) | <0.001 |
| Clusterin | 0.011 | (-0.065, 0.087) | | | 0.77 | 0.012 | (-0.062, 0.086) | | | 0.75 | 0.023 | (-0.108, 0.154) | 0.73 |
| Clusterin^2^ | -0.106 | (-0.208, -0.004) | | | 0.040 | -0.024 | (-0.122, 0.074) | | | 0.64 | -0.130 | (-0.304, 0.044) | 0.15 |
| Rs11136000 | 0.021 | (-0.104, 0.146) | | | 0.74 | 0.021 | (-0.101, 0.143) | | | 0.74 | 0.042 | (-0.174, 0.258) | 0.70 |
|  |  |  | | |  |  |  | | |  |  |  |  |
| Model 2 Intercept | 8.972 | (8.680, 9.264) | | | <0.001 | 8.785 | (8.503, 9.067) | | | <0.001 | 17.757 | (17.224, 18.290) | <0.001 |
| Clusterin | -0.061 | (-0.157, 0.035) | | | 0.21 | -0.062 | (-0.154, 0.030) | | | 0.19 | -0.123 | (-0.297, 0.051) | 0.17 |
| Clusterin^2^ | -0.147 | (-0.278, -0.016) | | | 0.028 | -0.064 | (-0.191, 0.063) | | | 0.33 | -0.211 | (-0.450, 0.028) | 0.084 |
| Rs9331888 | -0.127 | (-0.311, 0.057) | | | 0.18 | -0.111 | (-0.289, 0.067) | | | 0.22 | -0.238 | (-0.573, 0.097) | 0.17 |
|  |  |  | | |  |  |  | | |  |  |  |  |
| Model 3 Intercept | 8.881 | (8.638, 9.124) | | | <0.001 | 8.668 | (8.433, 8.903) | | | <0.001 | 17.549 | (17.130, 17.968) | <0.001 |
| Clusterin | 0.009 | (-0.067, 0.085) | | | 0.80 | 0.011 | (-0.063, 0.085) | | | 0.78 | 0.020 | (-0.111, 0.151) | 0.76 |
| Clusterin^2^ | -0.103 | (-0.205, -0.001) | | | 0.047 | -0.022 | (-0.120, 0.076) | | | 0.67 | -0.125 | (-0.299, 0.049) | 0.16 |
| Rs9331888 | -0.048 | (-0.193, 0.097) | | | 0.52 | -0.031 | (-0.172, 0.110) | | | 0.67 | -0.079 | (-0.330, 0.172) | 0.54 |
|  |  |  | | |  |  |  | | |  |  |  |  |
| **Black participants** |  | |  |  | |  | |  |  | |  |  |  |
| Model 1 Intercept | 8.346 | (8.132, 8.560) | | | <0.001 | 8.204 | (7.965, 8.443) | | | <0.001 | 16.550 | (16.127, 16.973) | <0.001 |
| Clusterin | -0.038 | (-0.201, 0.125) | | | 0.65 | -0.028 | (-0.210, 0.154) | | | 0.76 | -0.066 | (-0.389, 0.257) | 0.69 |
| Clusterin^2^ | -0.032 | (-0.312, 0.248) | | | 0.82 | 0.041 | (-0.273, 0.355) | | | 0.80 | 0.009 | (-0.550, 0.568) | 0.98 |
|  |  |  | | |  |  |  | | |  |  |  |  |
| Model 2 Intercept | 8.797 | (8.170, 9.424) | | | <0.001 | 8.596 | (7.890, 9.302) | | | <0.001 | 17.393 | (16.144, 18.642) | <0.001 |
| Clusterin | -0.051 | (-0.214, 0.112) | | | 0.54 | -0.040 | (-0.222, 0.142) | | | 0.67 | -0.091 | (-0.414, 0.232) | 0.58 |
| Clusterin^2^ | -0.014 | (-0.294, 0.266) | | | 0.92 | 0.056 | (-0.260, 0.372) | | | 0.73 | 0.042 | (-0.515, 0.599) | 0.88 |
| Rs9331888 | -0.278 | (-0.641, 0.085) | | | 0.14 | -0.241 | (-0.651, 0.169) | | | 0.25 | -0.519 | (-1.242, 0.204) | 0.16 |
|  |  |  | | |  |  |  | | |  |  |  |  |
| Model 3 Intercept | 9.019 | (8.488, 9.550) | | | <0.001 | 8.836 | (8.221, 9.451) | | | <0.001 | 17.856 | (16.833, 18.879) | <0.001 |
| Clusterin | 0.000 | (-0.131, 0.131) | | | >0.99 | 0.013 | (-0.138, 0.164) | | | 0.87 | 0.013 | (-0.238, 0.264) | 0.92 |
| Clusterin^2^ | -0.138 | (-0.371, 0.095) | | | 0.22 | -0.080 | (-0.333, 0.173) | | | 0.54 | -0.218 | (-0.639, 0.203) | 0.31 |
| Rs9331888 | -0.294 | (-0.580, -0.008) | | | 0.046 | -0.254 | (-0.585, 0.077) | | | 0.14 | -0.548 | (-1.099, 0.003) | 0.054 |
|  |  |  | | |  |  |  | | |  |  |  |  |
| Model 2 Intercept | 7.598 | (6.602, 8.594) | | | <0.001 | 7.338 | (6.223, 8.453) | | | <0.001 | 14.936 | (12.956, 16.916) | <0.001 |
| Clusterin | -0.059 | (-0.224, 0.106) | | | 0.48 | -0.052 | (-0.236, 0.132) | | | 0.58 | -0.111 | (-0.436, 0.214) | 0.51 |
| Clusterin^2^ | 0.002 | (-0.280, 0.284) | | | 0.99 | 0.081 | (-0.235, 0.397) | | | 0.62 | 0.083 | (-0.478, 0.644) | 0.77 |
| Rs113644261 | 0.386 | (-0.116, 0.888) | | | 0.14 | 0.447 | (-0.116, 1.010) | | | 0.12 | 0.832 | (-0.166, 1.830) | 0.11 |
|  |  |  | | |  |  |  | | |  |  |  |  |
| Model 3 Intercept | 7.582 | (6.782, 8.382) | | | <0.001 | 7.343 | (6.426, 8.260) | | | <0.001 | 14.926 | (13.397, 16.455) | <0.001 |
| Clusterin | -0.004 | (-0.133, 0.125) | | | 0.95 | 0.004 | (-0.145, 0.153) | | | 0.96 | 0.000 | (-0.247, 0.247) | >0.99 |
| Clusterin^2^ | -0.119 | (-0.339, 0.101) | | | 0.29 | -0.052 | (-0.303, 0.199) | | | 0.68 | -0.171 | (-0.588, 0.246) | 0.42 |
| Rs113644261 | 0.480 | (0.092, 0.868) | | | 0.017 | 0.543 | (0.098, 0.988) | | | 0.019 | 1.023 | (0.282, 1.764) | 0.008 |
|  |  |  | | |  |  |  | | |  |  |  |  |
| **White participants** |  | | | | | | | | | | | | |
| Model 1 Intercept | 8.926 | (8.793, 9.059) | | | <0.001 | 8.755 | (8.632, 8.878) | | | <0.001 | 17.681 | (17.446, 17.916) | <0.001 |
| Clusterin | -0.032 | (-0.146, 0.082) | | | 0.58 | -0.037 | (-0.143, 0.069) | | | 0.49 | -0.070 | (-0.272, 0.132) | 0.50 |
| Clusterin^2^ | -0.164 | (-0.307, -0.021) | | | 0.024 | -0.079 | (-0.210, 0.052) | | | 0.24 | -0.243 | (-0.496, 0.010) | 0.060 |
|  |  |  | | |  |  |  | | |  |  |  |  |
| Model 2 Intercept | 9.078 | (8.780, 9.376) | | | <0.001 | 8.914 | (8.638, 9.190) | | | <0.001 | 17.993 | (17.464, 18.522) | <0.001 |
| Clusterin | -0.038 | (-0.152, 0.076) | | | 0.52 | -0.043 | (-0.149, 0.063) | | | 0.43 | -0.081 | (-0.283, 0.121) | 0.43 |
| Clusterin^2^ | -0.163 | (-0.306, -0.020) | | | 0.025 | -0.078 | (-0.209, 0.053) | | | 0.25 | -0.241 | (-0.494, 0.012) | 0.063 |
| Rs11136000 | -0.120 | (-0.330, 0.090) | | | 0.26 | -0.124 | (-0.318, 0.070) | | | 0.21 | -0.244 | (-0.614, 0.126) | 0.20 |
|  |  |  | | |  |  |  | | |  |  |  |  |
| Model 3 Intercept | 8.956 | (8.686, 9.226) | | | <0.001 | 8.755 | (8.506, 9.004) | | | <0.001 | 17.711 | (17.260, 18.162) | <0.001 |
| Clusterin | 0.001 | (-0.091, 0.093) | | | 0.98 | 0.000 | (-0.086, 0.086) | | | >0.99 | 0.002 | (-0.153, 0.157) | 0.99 |
| Clusterin^2^ | -0.085 | (-0.199, 0.029) | | | 0.14 | -0.002 | (-0.108, 0.104) | | | 0.98 | -0.087 | (-0.279, 0.105) | 0.37 |
| Rs11136000 | -0.076 | (-0.245, 0.093) | | | 0.37 | -0.079 | (-0.234, 0.076) | | | 0.32 | -0.156 | (-0.436, 0.124) | 0.28 |
|  |  |  | | |  |  |  | | |  |  |  |  |
| Model 2 Intercept | 8.989 | (8.828, 9.150) | | | <0.001 | 8.807 | (8.656, 8.958) | | | <0.001 | 17.795 | (17.509, 18.081) | <0.001 |
| Clusterin | -0.031 | (-0.145, 0.083) | | | 0.60 | -0.036 | (-0.142, 0.070) | | | 0.50 | -0.067 | (-0.269, 0.135) | 0.51 |
| Clusterin^2^ | -0.175 | (-0.318, -0.032) | | | 0.017 | -0.088 | (-0.221, 0.045) | | | 0.20 | -0.263 | (-0.518, -0.008) | 0.044 |
| Rs17466684 | -0.184 | (-0.454, 0.086) | | | 0.18 | -0.150 | (-0.401, 0.101) | | | 0.24 | -0.334 | (-0.814, 0.146) | 0.17 |
|  |  |  | | |  |  |  | | |  |  |  |  |
| Model 3 Intercept | 8.944 | (8.762, 9.126) | | | <0.001 | 8.725 | (8.556, 8.894) | | | <0.001 | 17.669 | (17.365, 17.973) | <0.001 |
| Clusterin | 0.008 | (-0.084, 0.100) | | | 0.87 | 0.006 | (-0.078, 0.090) | | | 0.88 | 0.014 | (-0.139, 0.167) | 0.86 |
| Clusterin^2^ | -0.099 | (-0.213, 0.015) | | | 0.090 | -0.013 | (-0.119, 0.093) | | | 0.81 | -0.112 | (-0.302, 0.078) | 0.25 |
| Rs17466684 | -0.234 | (-0.448, -0.020) | | | 0.033 | -0.197 | (-0.395, 0.001) | | | 0.052 | -0.431 | (-0.788, -0.074) | 0.018 |
| Abbreviations: ECV, entorhinal cortex volume; HV, hippocampal volume; MTLV, medial temporal lobe volume; hsCRP, high sensitivity C-reactive protein.  ^a^ Based on 434 subjects with SNP, plasma clusterin, and MRI data.  ^b^Plasma clusterin was centered and standardized so that the beta coefficients from the models represent the following: ‘Intercept’ represents the mean MRI volume indicated (left column) when clusterin is equal to its mean; ‘Clusterin’ represents the slope of the association between clusterin and the MRI volume at mean clusterin; and ‘Clusterin^2^’ represents the change in the slope of the association between clusterin and MRI volume for each 1 SD difference in clusterin relative to its mean (see S2 Appendix for further details).  ^c^ Model 1: Intercept, clusterin, clusterin^2^.  ^d^ Model 2: Intercept, clusterin, clusterin^2^ and genetic variant (i.e. *Rs11136000*) previously found to be moderately associated with MRI markers in single SNP analysis (see S1 Appendix for further details).  ^e^ Model 3: Model 2 plus additional covariates including age, sex, race (included in non-stratified analysis), supratentorial brain volume, hsCRP. | | | | | | | | | | | | | |
