# Supplementary material for: Associations of plasma clusterin and Alzheimer’s disease-related MRI markers in adults at mid-life: The CARDIA Brain MRI sub-study
Source: PLoS One. 2018 Jan 11;13(1):e0190478. doi: 10.1371/journal.pone.0190478 (PMC5764276; doi:10.1371/journal.pone.0190478)
Supplement: S4 Table — (DOC) [file pone.0190478.s006.doc]

| **S4 Table. Associations of plasma clusterin^a^ and cognitive measures^b^** | | | | | |  | | | | | | | |  | | |
| --- | --- | --- | --- | --- | --- | --- | --- | --- | --- | --- | --- | --- | --- | --- | --- | --- |
| Unadjusted | | | | | Adjusted^c^ | | | | | | |  |  | | | |
|  | | Beta (95% CI) *P*-value | | | | |  | | Beta (95% CI) *P*-value | | | | | |  | |
| DSST Intercept | 0.040 | | (-0.052, 0.133) | 0.394 | | |  | 0.692 | | (0.560, 0.825) | <0.001 | | | | |  |
| Clusterin | 0.009 | | (-0.069, 0.087) | 0.826 | | |  | -0.009 | | (-0.080, 0.062) | 0.797 | | | | |  |
| Clusterin^2^ | -0.070 | | (-0.171, 0.031) | 0.117 | | |  | -0.049 | | (-0.139, 0.042) | 0.293 | | | | |  |
|  |  | |  |  | | |  |  | |  |  | | | | |  |
| RAVLT Intercept | -0.044 | | (-0.139, 0.051) | 0.359 | | |  | 0.559 | | (0.425, 0.694) | <0.001 | | | | |  |
| Clusterin | -0.010 | | (-0.090, 0.069) | 0.799 | | |  | -0.030 | | (-0.102, 0.042) | 0.412 | | | | |  |
| Clusterin^2^ | -0.007 | | (-0.110, 0.096) | 0.896 | | |  | 0.017 | | (-0.075, 0.109) | 0.717 | | | | |  |
|  |  | |  |  | | |  |  | |  |  | | | | |  |
|  | Odds Ratio | | (95% CI) | *P*-value | | |  | Odds Ratio | | (95% CI) | *P*-value | | | | |  |
| STROOP(2) Intercept | 0.899 | | (0.801, 1.010) | 0.360 | | |  | 0.583 | | (0.484, 0.703) | 0.004 | | | | |  |
| Clusterin | 1.005 | | (0.829,1.217) | 0.962 | | |  | 0.961 | | (0.784, 1.176) | 0.697 | | | | |  |
| Clusterin^2^ | 1.092 | | (0.853, 1.398) | 0.484 | | |  | 1.034 | | (0.800, 1.336) | 0.800 | | | | |  |
|  |  | |  |  | | |  |  | |  |  | | | | |  |
| STROOP(3) Intercept | 0.895 | | (0.796, 1.006) | 0.343 | | |  | 0.372 | | (0.303, 0.456) | 0.001 | | | | |  |
| Clusterin | 0.892 | | (0.734, 1.084) | 0.249 | | |  | 0.852 | | (0.688, 1.055) | 0.142 | | | | |  |
| Clusterin^2^ | 1.038 | | (0.801, 1.344) | 0.779 | | |  | 0.932 | | (0.703, 1.236) | 0.625 | | | | |  |
| Abbreviations: DSST, digit symbol substitution test; RAVLT, Reyes auditory-verbal learning test  ^a^Plasma clusterin was centered and standardized so that the beta coefficients from the models represent the following: ‘Intercept’ represents the mean cognitive measure (or odds ratio for STROOP) indicated (left column) when clusterin is equal to its mean; ‘Clusterin’ represents the slope of the association between clusterin and the cognitive measure at mean clusterin; and ‘Clusterin^2^’ represents the change in the slope of the association between clusterin and cognitive measure for each 1 SD difference in clusterin relative to its mean  ^b^DSST and RAVLT were standardized and represent z-scores. STROOP was categorized based on tertiles from an initial standardized z-score. Associations between STROOP and plasma clusterin are based on comparisons between the second (i.e., STROOP(2)) and third (i.e., STROOP(3)) tertiles with the first tertile (reference), respectively.  ^c^Adjusted for age, sex, race, supratentorial brain volume, hsCRP. Note: ‘Intercept’ in adjusted model represents mean cognitive measure (or odds ratio for STROOP) conditional on reference values of variables in model. | | | | | | | | | | | | | | | | |
